# Integrin α7 is overexpressed and correlates with higher pathological grade, increased T stage, advanced TNM stage as well as worse survival in clear cell renal cell carcinoma patients: A retrospective study

**DOI:** 10.1002/jcla.23034

**Published:** 2019-11-11

**Authors:** Yue Chen, Yang Wang, Zhixing Cai, Xiaoli Fan, Yi Zhang

**Affiliations:** ^1^ Department of TCM Tongren Hospital Shanghai Jiao Tong University School of Medicine Shanghai China

**Keywords:** clear cell renal cell carcinoma, clinicopathological characteristics, integrin α7, overall survival, prognosis

## Abstract

**Objective:**

This study aimed to explore the association of integrin α7 with clinicopathological characteristics and overall survival (OS) in clear cell renal cell carcinoma (ccRCC) patients.

**Methods:**

179 ccRCC patients who underwent nephrectomy were included in this retrospective study. Tumor tissue and paired adjacent tissue specimens of patients were obtained. Immunohistochemistry assay was performed to detect integrin α7 expression. OS was calculated with the median follow‐up duration of 91.0 months (range: 3.0‐116.0 months).

**Results:**

Integrin α7 was highly expressed in tumor tissue compared to paired adjacent tissue (*P* < .001), and tumor integrin α7 high expression was correlated with higher pathological grade (*P* = .004), increased T stage (*P* = .017), and advanced TNM stage (*P* = .033). Kaplan–Meier curve showed that patients with integrin α7 high expression (mean OS = 69.8, 95%CI: 60.5‐79.1 months) presented with worse OS compared to patients with integrin α7 low expression (mean OS = 101.8, 95%CI: 96.0‐107.7 months; *P* < .001). Multivariate Cox's regression analysis further disclosed that tumor integrin α7 high expression independently predicted poor OS (*P* < .001).

**Conclusion:**

Integrin α7 is upregulated and correlates with higher pathological grade, increased T stage, and advanced TNM stage, meanwhile it also acts as a valuable prognostic factor for worse survival in ccRCC patients.

## 1 INTRODUCTION

Renal cell carcinoma (RCC), the most common type of unitary malignancy, results in approximately 350 000 new cases and estimated 140 000 deaths per year worldwide.^1^ Clear cell RCC (ccRCC) is considered as the predominant histological subtype accounting for 70%‐80% of all RCC cases, which is insensitive to traditional radiotherapy or chemotherapy, leading to radical or partial nephrectomy to be optimized for ccRCC treatment.^1^, ^2^, ^3^ Although great effectiveness of surgical resection on removing tumor has been reported, there are still 20%‐40% ccRCC patients developing local recurrence or distant metastasis after surgery.^2^, ^4^ Hence, investigation of additional biomarkers to supervise disease development and progression is imperative in ccRCC patients.

Integrins, one family of cell adhesion molecules, are heterodimers consisting of an alpha and a beta subunit, which play important roles in many physiological and pathological processes via regulating cell‐cell or cell‐matrix adhesion.^5^ Integrin α7, belonging to the integrin family of adhesion molecule, is localized on chromosome 12p13 and consists of at least 27 exons spanning a region of about 22.5 kb.^6^ According to accumulating evidences, integrin α7 serves as an important regulator in tumor progression of several carcinomas, including prostate cancer, esophageal squamous cell carcinoma (OSCC), and glioblastoma.^5^, ^6^, ^7^, ^8^ In addition, integrin α7 also has been described as a functional cancer stem cells (CSCs) surface marker, and its knockdown could effectively decrease the stemness of cancer cells, such as OSCC, whereas the information about the role of integrin α7 in RCC, particular in ccRCC, is still rarely known.^6^ Considering the key role of integrin α7 in tumor progression and its effect on CSC properties in different carcinomas, we hypothesized that integrin α7 might play a critical role in ccRCC. Thus, this study aimed to explore the association of integrin α7 with clinicopathological characteristics and overall survival (OS) in ccRCC patients.

## 2 MATERIALS AND METHODS

### 2.1 Patients

Between Jan 2008 and Dec 2010, 179 ccRCC patients who underwent nephrectomy in Tongren Hospital of Shanghai Jiaotong University School of Medicine, Shuguang Hospital affiliated to Shanghai University of Traditional Chinese Medicine and Yueyang Hospital affiliated to Shanghai University of Traditional Chinese Medicine were included in this retrospective study. The inclusion criteria consisted of: (a) diagnosed as primary ccRCC; (b) age ≥18 years old; (c) tumor tissue sample and paired adjacent tissue were completely preserved and accessible; and (d) medical records and follow‐up data were complete and available. Following patients were excluded: (a) other types of RCC except for ccRCC, such as papillary, chromophobe, translocation, and Bellini duct (collecting duct) tumors; (b) secondary ccRCC; (c) with TNM stage IV or distant metastases; (d) had a history of malignant tumors other than ccRCC. The present study was approved by Institutional Review Board of Tongren Hospital Shanghai Jiao Tong University School of Medicine Hospital, and written informed consents were collected from all patients or their guardians.

### 2.2 Tissue specimens and data collection

Tumor tissue and paired adjacent tissue specimens of patients were obtained from Tumor Specimen storeroom of hospital. Baseline characteristics of patients were collected from medical records, which included age, gender, tumor location, pathological grade, tumor size, T stage, N stage, and TNM stage. In addition, survival data were also collected from patients’ follow‐up records, and the median follow‐up duration was 91.0 months (range: 3.0‐116.0 months).

### 2.3 Immunohistochemistry assay

Firstly, formalin‐fixed and paraffin‐embedded specimens of tumor tissue and paired adjacent tissue were serially sectioned at 4 μm thickness, and the sections were deparaffinized with xylene, rehydrated with ethanol, microwaved in 0.01 mol/L sodium citrate buffer (pH 6.0) for antigen retrieval, and incubated with H_2_O_2_ at 37°C for 10 minutes. Then 1.5% normal goat serum was used for blocking under 37°C for 20 minutes. Subsequently, the sections were incubated overnight with Rabbit polyclonal to ITGA7 antibody (1:200 dilutions; Abcam). Next, the sections were incubated with horseradish peroxidase‐conjugated goat anti‐rabbit immunoglobulin G antibody (diluted with 3% bovine serum albumin/PBS; 1:1000 dilutions; Abcam) incubated at 37°C for an hour. 3,3’‐Diaminobenzidine (DAB) and hematoxylin were used as chromogenic agents. After sealed with neutral tree gum sequentially, sections were prepared for visualization under microscope (Olympus). After staining, five randomly selected areas were evaluated in every tissue slide under the microscope (200 × original magnification). The intensity of positively stained cells was scored. After that, the numbers of positively stained cells were counted, and then, the percentages of positively stained cells were calculated and scored. The average intensity score and proportion score were used to calculate the total score. Two investigators blinded to the type of tissues performed these procedures. For staining in tumor tissue and paired adjacent tissue, it was visually scored and quantified according to a semiquantitative scoring system.^9^ Briefly, the intensity of immunostaining was scored as: 0 = negative (no staining), 1 = weak (light yellow), 2 = moderate (yellow), and 3 = strong (brown); and the proportion of immune‐positive cells was scored as: 1 = <10%, 2 = 10% ~ 50%, 3 = 51% ~ 75%, and 4 = more than 75%. The two scores of the corresponding sample were multiplied to obtain a total score ranging from 0 to 12 points. High expression of integrin α7 was defined as total score ≥3, and low expression of integrin α7 was defined as total score <3. All procedures in this experiment were performed by one specialist from the Laboratory Department of our hospital who were blinded to the type of tissues and patients’ information. In addition, the IHC was scored by two clinic pathologists with rich experience from the Pathological Department of our hospital who were blinded to the type of tissues and patients’ information as well. When the opinion of these two clinic pathologists was different, the third clinic pathologist with rich experience from the Pathological Department of our hospital who were blinded to the type of tissues and patients’ information would score the results of IHC.

### 2.4 Statistical analysis

Statistical analysis was performed by SPSS 19.0 software (IBM), and graph making was accomplished using GraphPad Prism 7.00 (GraphPad Inc). Normal distributed continuous variables were presented as mean value ± standard deviation; skewed distributed continuous variables were presented as median (25th‐75th quantiles); and categorized variables were presented as count (percentage). Comparison of percentage was determined by Chi‐square test or Wilcoxon rank sum test. Survival analysis was performed using Kaplan–Meier (K‐M) curve and Log‐rank test. Factors affecting OS were determined by univariate and multivariate Cox's proportional hazards regression analyses with Forward Stepwise (Conditional) method. *P* value < .05 was considered as significant.

## 3 RESULTS

### 3.1 Study flow

**Figure 1:**
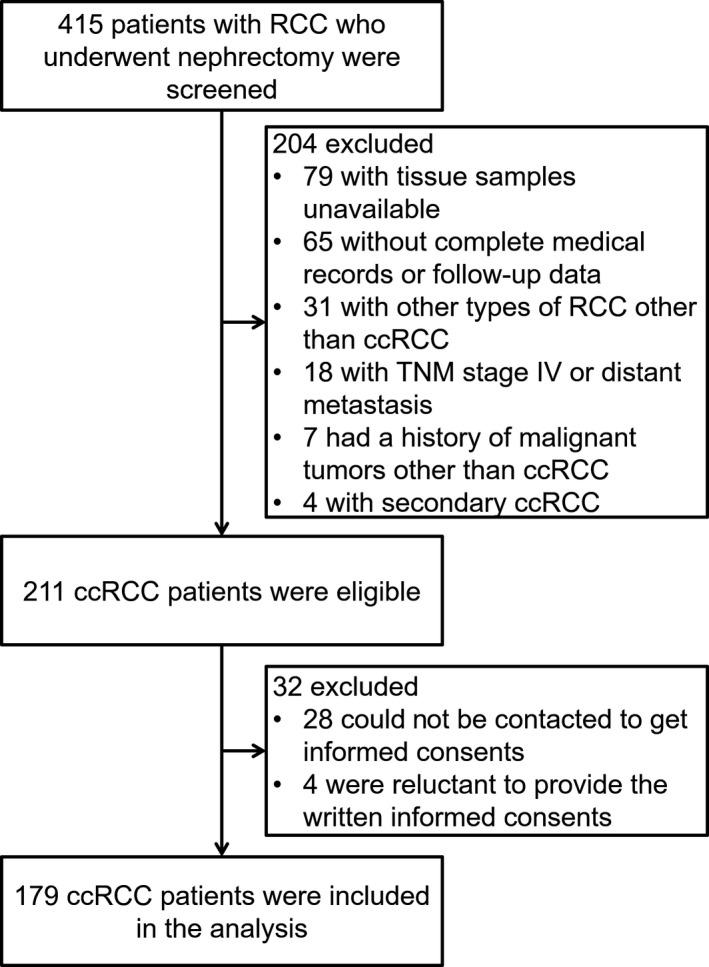
Study flow

A total of 415 patients with RCC who underwent nephrectomy were screened, while 204 cases were excluded, among which 79 cases were with tissue samples unavailable, 65 cases were without complete medical records or follow‐up data, 31 cases were with other types of RCC other than ccRCC, 18 cases were with TNM stage IV or distant metastasis, seven cases had a history of malignant tumors other than ccRCC, and four cases were with secondary ccRCC (Figure 1). Subsequently, 211 ccRCC cases were eligible, whereas 32 cases were excluded, including 28 cases who could not be contacted to get informed consents and four cases who were reluctant to provide the written informed consents. Finally, the remaining 179 ccRCC cases were included in the analysis.

### 3.2 Baseline characteristics

The mean age of 179 included ccRCC patients was 59.8 ± 12.3 years, and there were 102 male (57.0%) and 77 females (43.0%; Table 1)**.** The number of patients with pathological grade 1, 2, and 3 was 79 (44.1%), 78 (43.6%), and 22 (12.3%), respectively. The median tumor size was 5.0 (4.0‐7.5) cm. In addition, 125 (69.8%), 36 (20.1%), and 18 (10.1%) patients were with TNM stage I, II, and III. Other characteristics were shown in Table 1.

**Table 1:** Baseline characteristics of ccRCC patients

Items	ccRCC patients (N = 179)
Age (y)	59.8 ± 12.3
Gender	
Male (n/%)	102 (57.0)
Female (n/%)	77 (43.0)
Tumor location	
Right (n/%)	86 (48.0)
Left (n/%)	93 (52.0)
Pathological grade	
1 (n/%)	79 (44.1)
2 (n/%)	78 (43.6)
3 (n/%)	22 (12.3)
Tumor size (cm)	5.0 (4.0‐7.5)
T stage	
T1 (n/%)	131 (73.2)
T2 (n/%)	38 (21.2)
T3 (n/%)	10 (5.6)
N stage	
N0 (n/%)	167 (93.3)
N1 (n/%)	12 (6.7)
TNM stage	
I (n/%)	125 (69.8)
II (n/%)	36 (20.1)
III (n/%)	18 (10.1)

Note

Data were presented as mean value ± standard deviation, count (percentage), or median (25th‐75th quantiles). Pathological grade 1: well differentiated; Pathological grade 2: moderately differentiated; Pathological grade 3: poorly differentiated. ccRCC: clear cell renal cell carcinoma.

### 3.3 Expression of integrin α7 in tumor tissue and paired adjacent tissue

**Figure 2:**
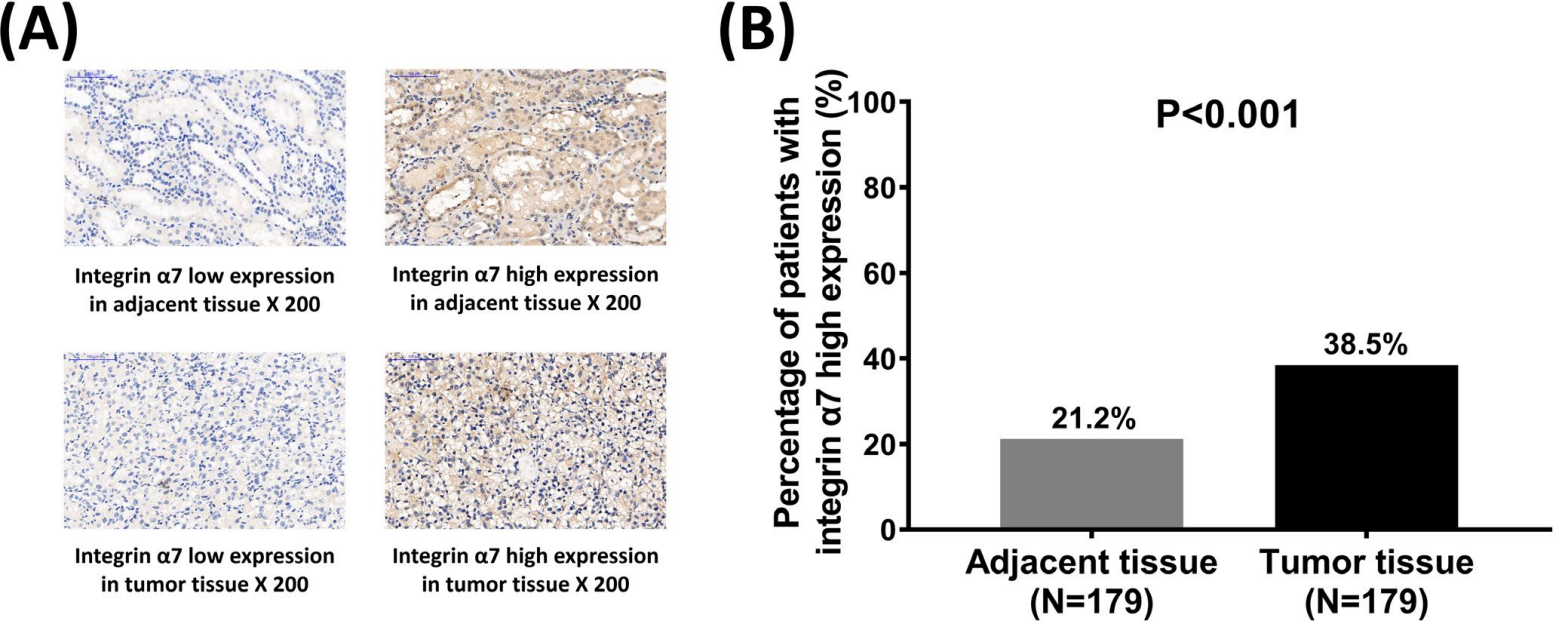
Integrin α7 expression between tumor tissue and adjacent tissue. Immunohistochemistry assay revealed that integrin α7 was highly expressed in tumor tissue (A). The percentage of patients with integrin α7 high expression in tumor tissue was increased than that in adjacent tissue (B). Comparison was determined by Chi‐square test. *P* < .05 was considered as significant

Immunohistochemistry assay was used to detect the expression of integrin α7 in tumor tissue and paired adjacent tissue, and examples of integrin α7 high or low expression assessed by semiquantitative scoring were shown in Figure 2A. Most importantly, integrin α7 expression was observed to be increasingly expressed in tumor tissue compared to paired adjacent tissue (*P* < .001; Figure 2B).

### 3.4 Association of tumor integrin α7 expression with tumor characteristics

Tumor integrin α7 high expression was correlated with higher pathological grade (*P* = .004), increased T stage (*P* = .017), and advanced TNM stage (*P* = .033; Table 2). However, no correlation was found between tumor integrin α7 expression and other characteristics (All *P* > .05).

**Table 2:** Correlation of tumor integrin α7 expression with tumor characteristics

Characteristics	Tumor integrin α7 expression		*P* value
	High	Low	
Tumor location			.333
Right (n/%)	30 (34.9)	56 (65.1)	
Left (n/%)	39 (41.9)	54 (58.1)	
Pathological Grade			**.004**
1 (n/%)	24 (30.4)	55 (69.6)	
2 (n/%)	29 (37.2)	49 (62.8)	
3 (n/%)	16 (72.7)	6 (27.3)	
Tumor size			.107
≤5 cm (n/%)	31 (33.0)	63 (67.0)	
>5 cm (n/%)	38 (44.7)	47 (55.3)	
T stage			**.017**
T1 (n/%)	44 (33.6)	87 (66.4)	
T2 (n/%)	18 (47.4)	20 (52.6)	
T3 (n/%)	7 (70.0)	3 (30.0)	
N stage			.146
N0 (n/%)	62 (37.1)	105 (62.9)	
N1 (n/%)	7 (58.3)	5 (41.7)	
TNM stage			**.033**
I (n/%)	42 (33.6)	83 (66.4)	
II (n/%)	17 (47.2)	19 (52.8)	
III (n/%)	10 (55.6)	8 (44.4)	

Note

Data were presented as count (percentage). Comparison was determined by Chi‐square test or Wilcoxon rank sum test. *P* value < .05 was considered significant (in bold). Pathological grade 1: well differentiated; Pathological grade 2: moderately differentiated; Pathological grade 3: poorly differentiated.

### 3.5 Correlation of tumor integrin α7 expression with OS

**Figure 3:**
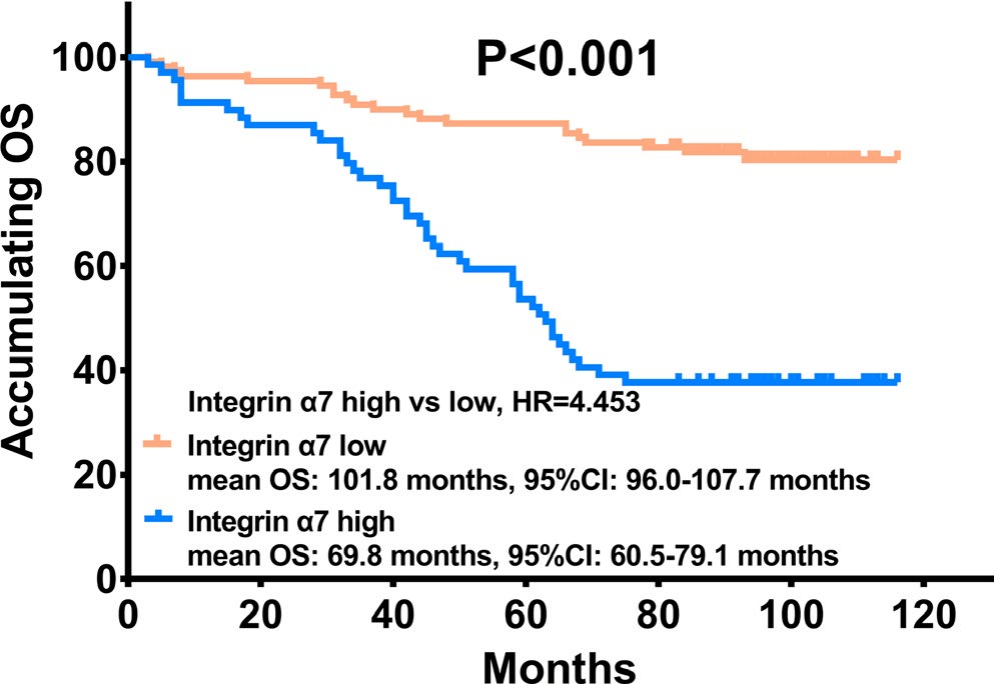
Association between tumor integrin α7 and OS. Tumor integrin α7 high expression was correlated with worse OS in ccRCC patients. Kaplan–Meier curve and Log‐rank test were used for survival analysis. *P* < .05 was considered as significant. OS: overall survival

K‐M curve showed that mean OS was 101.8 months (95%CI: 96.0‐107.7 months) in integrin α7 low expression group and 69.8 months (95%CI: 60.5‐79.1 months) in integrin α7 high expression group (Figure 3). Tumor integrin α7 high expression was correlated with worse OS in ccRCC patients (*P* < .001).

### 3.6 Factors influencing OS in ccRCC patients

Univariate Cox's regression revealed that tumor integrin α7 high expression (*P* < .001), age (>60 years vs ≤60 years; *P* = .001), higher pathological grade (*P* < .001), tumor size (>5 cm vs ≤5 cm; *P* = .006), higher T stage(*P* < .001), N stage (1 vs 0; *P* < .001), and higher TNM stage (*P* < .001) were associated with shorter OS (Table 3). Multivariate Cox's regression with Forward Stepwise (Conditional) method was further performed, which disclosed that tumor integrin α7 high expression (*P* < .001), higher pathological grade (*P* < .001), and higher TNM stage (*P* < .001) could independently predict poor OS in ccRCC patients.

**Table 3:** Factors affecting OS by Cox's proportional hazards regression analysis

Items	Cox's regression model			
	*P* value	HR	95% CI	
			Lower	Higher
Univariate Cox's regression				
Tumor integrin α7 high expression	**<.001**	4.453	2.633	7.532
Age (>60 y vs ≤60 y)	**.001**	2.446	1.451	4.125
Gender (male vs female)	.538	0.857	0.525	1.400
Tumor location (left vs right)	.815	1.061	0.649	1.733
Higher pathological grade	**<.001**	2.915	2.012	4.222
Tumor size (>5 cm vs ≤5 cm)	**.006**	2.041	1.229	3.389
Higher T stage	**<.001**	2.859	1.991	4.106
N stage (1 vs 0)	**<.001**	8.197	4.297	15.636
Higher TNM stage	**<.001**	3.160	2.308	4.328
Multivariate Cox's regression with Forward Stepwise (Conditional) method				
Tumor integrin α7 high expression	**<.001**	3.353	1.900	5.918
Higher pathological grade	**<.001**	2.130	1.407	3.224
Higher TNM stage	**<.001**	3.667	2.382	5.644

Note

Factors affecting OS were determined by univariate and multivariate Cox's proportional hazards regression analyses with Forward Stepwise (Conditional) method. *P* value < .05 was considered significant (in bold). CI, confidence interval; HR, hazard ratio; OS, overall survival.

## 4 DISCUSSION

In the present study, we observed that (1) Integrin α7 was highly expressed in tumor tissue, and its high expression was associated with advanced cancer features. (2) Tumor integrin α7 high expression independently predicted poor OS in ccRCC patients.

Integrin α7, a member of the extracellular matrix binding proteins, contributes to the interaction of relevant cell‐cell and cell‐extracellular matrix in a wide range of cellular processes, which also involves in the processes of tumorigenesis and tumor progression in different malignancies.^5^, ^7^ A majority of previous studies have focused on the function of integrin α7 on cell activities in different carcinomas and disclosed its tumor promoter role in these cancers. For example, integrin α7 interacts with S100P to promote cells migration and cells invasion in lung carcinoma.^10^ Another mechanistic study discloses that integrin α7 induces cells migration and invasion abilities via the activation of epithelial–mesenchymal transition (EMT) in OSCC.^6^ In addition, parts of previous studies have revealed that integrin α7 possesses effect on regulating stemness of cancer cells. For instance, integrin α7 effectively promotes the stemness of OSCC cells through regulating the focal adhesion kinase (FAK)‐mediated pathway in OSCC.^6^ Therefore, these previous evidences suggest that integrin α7 appears to be a promoter in the pathological processes of several carcinomas due to its effect on cell activities and stemness of cancer cells.

In clinical trials, there is limited information about the association of integrin α7 with disease conditions in carcinomas, among which one previous study reveals that integrin α7 is related to poor differentiation and lymph node metastasis in OSCC patients, while little is known about the participation of integrin α7 in RCC patients, particularly ccRCC patients.^6^ In consideration of the underlying mechanism and influence of integrin α7 in different carcinomas as well as its correlation with the stemness of cancer cells, we suspected that integrin α7 might exert an influence on tumor progression in ccRCC patients. Thus, we evaluated the association of integrin α7 with clinicopatholgocial features in ccRCC patients, and we found that integrin α7 was highly expressed in tumor tissue, and its high expression was associated with higher pathological grade, increased T stage, and advanced TNM stage in ccRCC patients, which might have resulted from that: Firstly, integrin α7 contibuted to the activation of cell viabilities, including cell proliferation, differentiation, migration, or invasion, through inducing several onco‐genesis signaling pathways (including EMT pathway), thereby promoting tumor growth and metastasis, thereby resulting in advanced tumor features in ccRCC patients. Secondly, integrin α7 was involved in stemness regulation and CSC maintenance via inducing several signal pathways (such as FAK/MAPK/ERK pathway) to strengthen abilities of self‐renew, cell differentiation and cell motility, subsequently resulted in advanced clinical stage and metastasis, thereby caused worse disease conditions in ccRCC patients.

As for the prognostic value of integrin α7 in carcinomas, some studies illustrate that it correlates with better metastasis‐free survival in prostate cancer patients as well as soft tissue leiomyosarcoma patients, while another previous study displays that integrin α7 is associated with worse OS in OSCC patients as well as glioblastoma patients.^6^, ^7^, ^11^, ^12^ Therefore, there are controversial results related to the prognostic value of integrin α7 in patients with different carcinoma, which might be caused by different inclusion and exclusion criteria or different sample size and its function in different carcinomas. In the present study, we discovered that integrin α7 could independently predict shorter OS in ccRCC patients. The possible reasons were as follows: (a) Integrin α7 devoted in the exacerbation of disease conditions through increasing tumor growth and metastasis by mediating some pathways (such as EMT pathway), thereby led to shorter survival in ccRCC patients. (b) Integrin α7 might regulate multiple pathways (including the corresponding FAK/MAPK/ERK pathway) to increase stemness of cancer cells, subsequently enhancing the abilities to self‐renew, differentiate and chemoresistance, which caused poor survival in ccRCC patients.

Although some interesting results were discovered in this study, some limitations still existed. Firstly, this is a retrospective study, and we screened all patients who had completed records regarding OS, but not DFS, and the effect of integrin α7 on DFS in ccRCC patients was not investigated due to the loss of DFS records. Secondly, the sample size in this study was relatively small, which might cause poor statistical power. Thirdly, all patients enrolled in this study were from our hospital, which might result in regional bias.

In a word, integrin α7 is upregulated in tumor tissue, and it correlates with higher pathological grade, increased T stage, and advanced TNM stage; meanwhile, it also acts as a valuable prognostic factor for worse survival in ccRCC patients.

## CONFLICT OF INTEREST

The authors declare that they have no conflicts of interest.
